# Global Research Trends of Artificial Intelligence on Histopathological Images: A 20-Year Bibliometric Analysis

**DOI:** 10.3390/ijerph191811597

**Published:** 2022-09-15

**Authors:** Wentong Zhou, Ziheng Deng, Yong Liu, Hui Shen, Hongwen Deng, Hongmei Xiao

**Affiliations:** 1 Center for System Biology, Data Sciences, and Reproductive Health, School of Basic Medical Science, Central South University, Changsha 410031, China; 2 Tulane Center of Biomedical Informatics and Genomics, Deming Department of Medicine, School of Medicine, Tulane University School, New Orleans, LA 70112, USA

**Keywords:** artificial intelligence, histopathological images, bibliometrics, CiteSpace, VOSviewer

## Abstract

Cancer has become a major threat to global health care. With the development of computer science, artificial intelligence (AI) has been widely applied in histopathological images (HI) analysis. This study analyzed the publications of AI in HI from 2001 to 2021 by bibliometrics, exploring the research status and the potential popular directions in the future. A total of 2844 publications from the Web of Science Core Collection were included in the bibliometric analysis. The country/region, institution, author, journal, keyword, and references were analyzed by using VOSviewer and CiteSpace. The results showed that the number of publications has grown rapidly in the last five years. The USA is the most productive and influential country with 937 publications and 23,010 citations, and most of the authors and institutions with higher numbers of publications and citations are from the USA. Keyword analysis showed that breast cancer, prostate cancer, colorectal cancer, and lung cancer are the tumor types of greatest concern. Co-citation analysis showed that classification and nucleus segmentation are the main research directions of AI-based HI studies. Transfer learning and self-supervised learning in HI is on the rise. This study performed the first bibliometric analysis of AI in HI from multiple indicators, providing insights for researchers to identify key cancer types and understand the research trends of AI application in HI.

## 1. Introduction

Cancer is a widespread disease that causes major death in the world. In 2020, more than 19 million cases of cancer were confirmed worldwide, including over 10 million deaths from cancer [1]. The early diagnosis and treatment for cancer are essential to reduce cancer deaths in the world. Histopathological diagnosis is the gold standard for tumor diagnosis, grade, and classification. Although CT, MRI, and other imaging methods for tumor examination are provided clinically, cancer can only be diagnosed by biopsy and histopathological examination. Therefore, accurate histopathological diagnosis is critical for cancer screening and diagnosis. Histopathological examination is a highly subjective task that relies on professional knowledge and experience to evaluate tumor tissue structure and cell morphology in pathological sections. Unfortunately, pathologists with highly professional experience are often in short supply. Objective and accurate artificial intelligence (AI) histopathological methods could reduce the clinical misdiagnosis of histopathology caused by subjectivity and the lack of professionals [2].

AI is a technology that utilizes computer technology and algorithms to imitate human intelligence. With the rapid development of computer technology, AI-based machine learning and deep learning methods have been widely used in image processing [3]. As a data-driven approach, AI learns features from datasets relevant to downstream tasks. Digital pathology technology enables large-scale digitization of histopathological slices, and the massive growth of data of histopathological images (HI) accelerates the rapid development of AI in this field. AI has been used in early cancer diagnosis [4,5], cancer mutation detection [6,7], nuclear segmentation [8,9], prognosis evaluation [10,11], etc. The combination of AI and pathologists is a conducive way to solve the lack of professional pathologists in some remote regions [12,13,14]. A large number of papers have been published on AI applications in the fields of HI, so it is worthwhile to quantitatively analyze the papers in this field to grasp the research status and research trends as a whole.

Bibliometrics is a method used to quantitatively analyze numerous documents in a certain field. By counting the authors, countries, journals, and citation relationships of published literature in the field, the bibliometrics can visualize the connection and structure of research topics among the literature to determine current research hotspots and future development trends [15]. There are many studies reviewed on the topic of AI in HI [16,17,18,19], but only one bibliometric study about breast cancer in histopathological image classification [20]. The study is limited to the classification task and breast cancer types. It is necessary to conduct a bibliometric analysis of multi-cancer and multi-tasks on the application of AI in HI. The research themes and emerging trends can be objectively displayed in the field through quantitative analysis of the literature data.

This study is the first bibliometric study to provide a comprehensive view of the application of AI in HI. We collected the publications from 2001 to 2021 in the Web of Science collection database, then counted and visualized multiple indicators by VOSviewer and CiteSpace. This study summarized the prevailing topics of cancer types, methods, and development trends of AI in HI, and provides a reference for future researchers to grasp key points.

## 2. Materials and Methods

### 2.1. Search Methods

A total of 6 Citation Indexes were used to ensure the comprehensiveness of the literature data from the Web of Science Core Collection (WOSCC) database, which are Science Citation Index Expanded (SCI-Expanded), Social Sciences Citation Index (SSCI), Conference Proceedings Citation Index-Science (CPCI-S), Arts & Humanities Citation Index (A & HCI), Emerging Sources Citation Index (ESCI), and Conference Proceedings Citation Index-Social Sciences & Humanities (CPCI-SSH). We used the Exact search function to collect articles, proceedings papers, and review articles from 2001 to 2021 on 5 May 2022 to prevent data update. The “plain text” format file with “Full Record and Cited References” was exported through WOSCC, which contains the titles, abstract, authors, keywords, journals, and reference records of the collected literature data. 

Because most keywords of AI in HI are terminology, we tried to include various methodological terminology [21]. We used the search rules as follows: TS = (“deep learning” OR “artificial intelligence” OR “machine learning” OR “feature extraction” OR “intelligent learning” OR “feature extraction” OR “feature mining” OR “feature embedding” OR “instance segmentation” OR “Semantic segmentation” OR “image* segmentation” OR superpixel OR “data mine” OR “neural network” OR “deep network” OR “neural learning” OR “neural nets model” OR “artificial neural network” OR “deep neural network” OR “Convolutional Neural Networks” OR CNN OR “supervised learning” OR “semi-supervised” OR “unsupervised learning” OR “unsupervised clustering” OR adversarial generative OR “Bayes network” OR self-supervised OR “active learning” OR “few-shot learning” OR “continual learning” OR “transfer learning” OR “domain adaptation” OR “metric learning” OR “contrastive learning” OR “reinforcement learning” OR “meta learning” OR “knowledge graph” OR “graph learning” OR “graph mining” OR SVM) AND TS = (‘histopatholo* image*’ OR “whole slide image*” OR “slide image*” OR “digital pathology”).

### 2.2. Bibliometric Analysis Methods

Next, we focused on analyzing the indicators such as country, region, institution, author, keywords, and references collected by the above retrieval methods. Co-authorship analysis can reflect the cooperative relationship between countries, authors, and institutions in the literature data. Co-occurrence shows the relationship between documents by quantifying the terms in the bibliographic data. Co-citation is a method to explore patterns in influential literature and the structural characteristics of current documents [22].

In this study, we used statistical analysis and visual analysis by using VOSviewer (version 1.6.18, Leiden University, Leiden, The Netherlands) [23], CiteSpace (version 5.8.R3, Drexel University, Philadelphia, PA, USA) [24], and Microsoft EXCEL. VOSviewer was used for the co-authorship analysis of countries/regions, authors, institutions, and author keywords. CiteSpace was used for the co-citation reference analysis and timeline visualization, and the log-likelihood ratio was used as the algorithm for obtaining cluster theme markers. The co-citation network used the Modularity Q value and the Weighted Mean Silhouette S value to measure the clustering effect and network homogeneity, respectively. The higher the two indicators, the more reasonable and effective the network clustering effect is [25,26]. A double-graph overlay of the journals was drawn [27] to analyze the distribution of citation trajectories and literature information. The impact factor (IF) is used to evaluate the quality of the journals. The higher the IF of a journal, the higher the quality of the literature published in that journal. The IF statistics are from the 2020 impact factors of the Web of Science core database. In addition, the visualization of the numbers of publications by different countries/regions was plotted using python3.6 (Guido van Rossum, Amsterdam, Netherlands). Microsoft EXCEL was used for statistical chart analysis and production.

## 3. Results

### 3.1. Global Publications Trends

**Figure 1:**
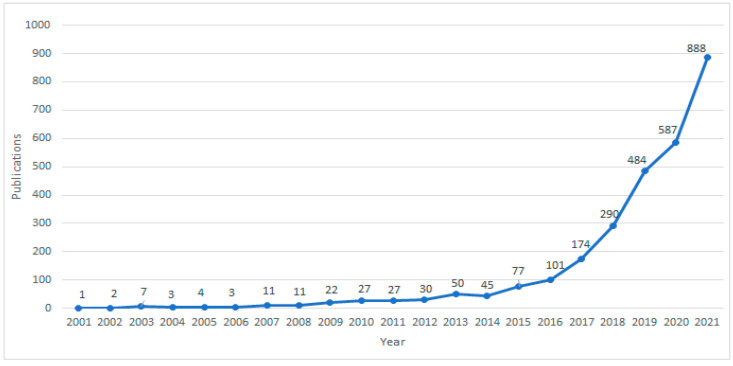
Global Publication Trends on AI in HI from 2001 to 2021.

Using the retrieval method described in Methods, a total of 2844 papers were retrieved, including 1714 articles, 945 proceedings papers, and 185 review articles. We found that the number of AI studies on pathological images has shown explosive growth in the past five years from 174 to 888 (Figure 1), accounting for 85.2% of the total number of publications, indicating that pathological AI analysis has become a hot research topic in recent years.

### 3.2. Countries and Regions

**Figure 2:**
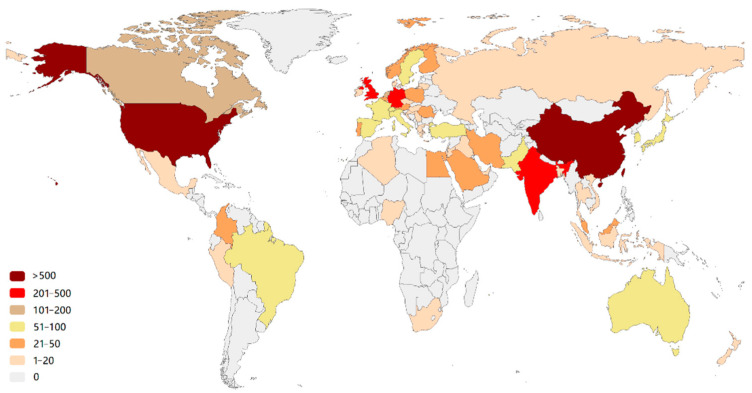
Country production map based on the total publications. The color indicates the number of publications.

**Figure 3:**
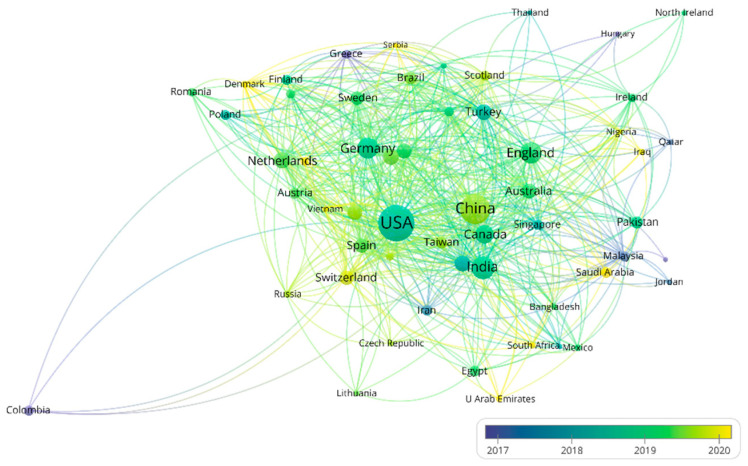
The network of countries/regions’ co-authorship analysis. Node color and size indicate average publication year and the number of publications, respectively; thickness of lines indicates the strength of the relationship. Each country/region in this map has at least 5 collaboration publications. The average year of publications in different countries are from 2017 to 2020.

More than 58 countries around the world have participated in the research on the AI application of HI (Figure 2). The top 10 most productive countries and regions are shown in Table S1. The USA is the most productive (the number of publications is 937) and influential (the number of citations is 23,010) country, followed by China (550 publications and 6631 citations). Notably, some countries (e.g., the Netherlands and France) are associated with a relatively high number of citations but with a modest number of publications. This is largely due to a few highly cited review articles, e.g., the article “A survey on deep learning in medical image analysis” by Litjens et al. [28] from the Netherlands (4078 citations). The visualization of the co-authorship analysis of countries is shown in Figure 3. The AI-based research in HI analysis in the USA was carried out as early as 2001 [29], while in China it began in 2009 [30]. The USA has cooperative relations with many countries. It is at the center of the network with the highest total link strength (total link strength is 731). Less intense cooperation occurs among other countries.

### 3.3. Institutions

**Figure 4:**
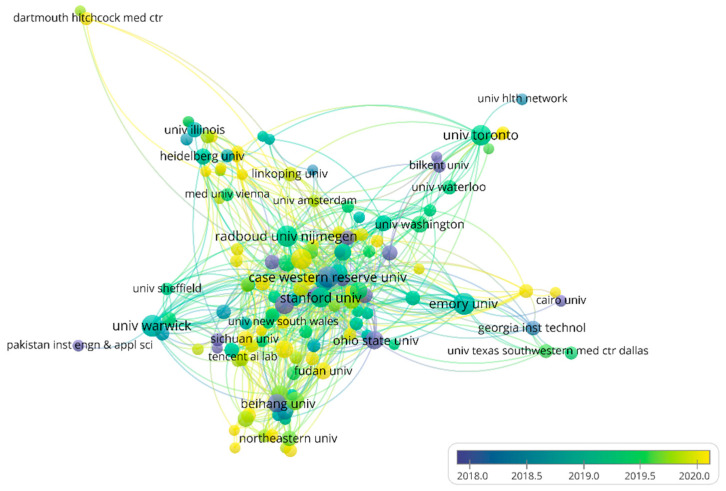
The network of institutions’ co-authorship analysis. Node color and size indicate the average publication year and the number of publications respectively; thickness of lines indicates the strength of the relationship. Each institution in this map has at least 10 collaborative publications. The average years of publications in the institutions are from 2018 to 2020.

About 3160 institutions have participated in the application of AI in HI. The top 10 most productive institutions are shown in Table S2, among which six institutions are from the United States. The top two institutions are Emory University and The University of Warwick with 55 and 54 articles, respectively. Emory University has mainly focused on AI combined with genomics to analyze HI [31,32,33], while the University of Warwick has focused on segmentation in HI [34,35,36]. Radboud University Nijmegen (citations = 6316) and Stanford University (citations = 5423) are the two institutions affiliated with the most citations. In particular, a review of “A survey on deep learning in medical image analysis” by Litjens et al. [28] (citations = 4078) and a skin cancer classification study “Dermatologist-level classification of skin cancer with deep neural networks” by Esteva et al. [37] (citations = 4273) contributed most of the citations in the two institutions, respectively. The co-authorship between institutions is shown in Figure 4. Most of the sub-networks contain institutions from the same countries, indicating that institutions from the same countries are more likely to collaborate, which is not unexpected. Most of the institutions in the center of the network are from the USA, indicating that increased cooperation is conducive to publishing more papers.

### 3.4. Authors

**Figure 5:**
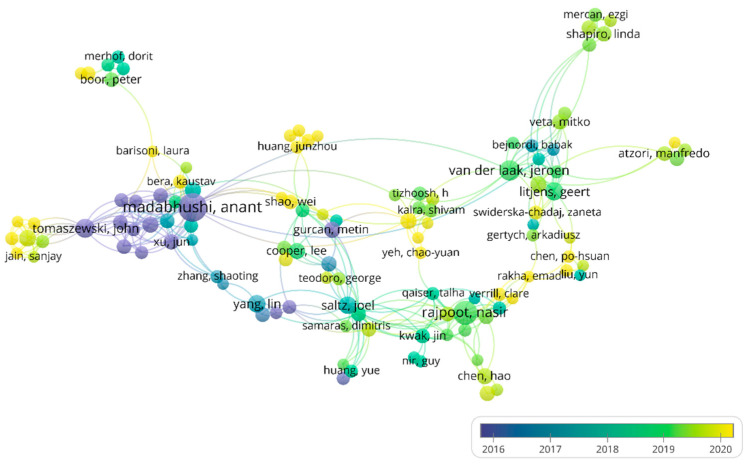
The network of authors’ co-authorship analysis. Node color and size indicate the average publication year and the number of publications, respectively; thickness of lines indicates the strength of the relationship. Each author in this map has at least 7 collaborative publications. The average years of publications of authors were from 2016 to 2020.

The top 10 most productive authors were all from Western countries, including half from the USA (Table S3). Dr. Anant Madabhushi is the most prolific author with 72 publications, having started early in the analysis of histopathology artificial intelligence in breast cancer [38,39,40,41]. The co-authorship analysis between authors is shown in Figure 5. There is a sub-network formed around Dr. Madabhushi, indicating that collaboration can promote research advancement. The multiple yellow nodes in Figure 5 indicate that more researchers are becoming devoted to AI in HI analysis, which shows that the application of AI in HI holds great promise.

### 3.5. Journals

**Figure 6:**
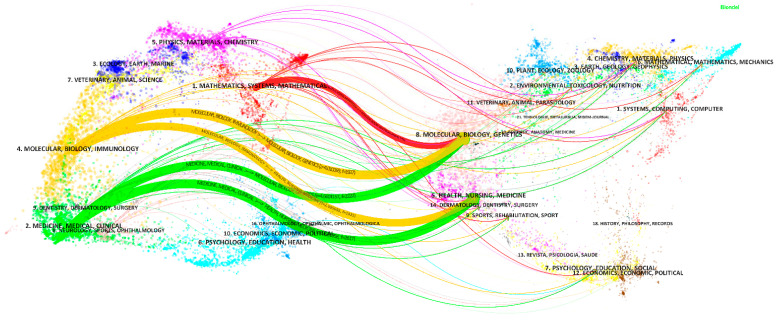
Dual-map overlay of journals on the application of AI in HI. The width of the paths is proportional to the z-score-scaled citation frequency. From top to bottom, the content in the main paths are: MATHEMATICS, SYSTEMS, MATHEMATICAL → MOLECULAR, BIOLOGY, GENETICS (z = 1.84, f = 986); MOLECULAR, BIOLOGY, IMMUNOLOGY → MOLECULAR, BIOLOGY, GENETICS (z = 4.01, f = 1937); MOLECULAR, BIOLOGY, IMMUNOLOGY → HEALTH, NURSING, MEDICINE (z = 2.60, f = 1321); MEDICINE, MEDICAL, CLINICAL → MOLECULAR, BIOLOGY, GENETICS (z = 4.66, f = 2222); and MEDICINE, MEDICAL, CLINICAL → HEALTH, NURSING, MEDICINE (z = 5.56, f = 2617).

The top 10 journals with the largest number of publications are shown in Table S4. Scientific Reports has the largest number of published papers. The two most cited journals are Medical Image Analysis and IEEE Transactions on Medical Imaging, which have the high IF (8.545 and 10.048, respectively). The high IFs and the high numbers of citations indicate that in the application of AI in HI, the two journals might have high quality and influence. Figure 6 is a dual-map overlay of journals on AI-related research on HI, with the citing journal map on the left and the cited journal map on the right. The citing papers were mainly distributed in three areas: (1) Mathematics, Systems, Mathematical; (2) Molecular, Biology, Immunology; (3) Medicine, Medical, Clinical. The cited papers were mainly distributed in two areas: (4) Molecular, Biology, Genetics; (5) Health, Nursing, Medicine. There are five main citation paths from the dual map, showing that the current application of AI in HI has been mainly published in disciplinary areas (1), (2), and (3), the main citation is from disciplinary areas (4) and (5).

### 3.6. Keywords

**Figure 7:**
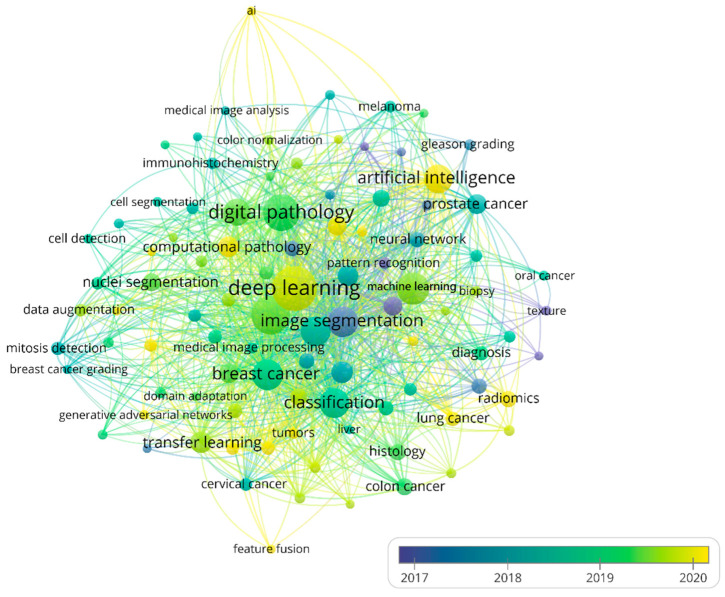
The network of author keywords’ co-occurrence analysis. Node color and size indicate average publication year and the number of occurrences, respectively; thickness of lines indicates the strength of the relationship. The occurrences of each keyword in this map were at least 11 times. The average years of the publications with the keywords were from 2017 to 2020.

We summarize the top 10 most frequent keywords of methods and cancer types to identify hotspots of AI in HI (Table S5). “Deep learning” had the highest occurrences (*n* = 657) in the method keywords, followed by “convolutional neural network” (*n* = 445), which was the main image analysis method in deep learning. Image data is often high-dimensional, and deep learning automatically and efficiently extracts features from images, which are often adapted to downstream tasks without feature engineering [42]. The top four cancer keywords with the most occurrences (*n*) were “breast cancer” (*n* = 298), “prostate cancer” (*n* = 90), “colorectal cancer” (*n* = 55), and “lung cancer” (*n* = 45), indicating that these cancers are hotspots in current research. The reason for that may be that breast cancer and lung cancer are the cancers with the highest mortality rates in the United States and China, respectively, and prostate cancer and colorectal cancer have shown rapidly increased incidence rates in recent years [43]. The three least-occurring cancer types with occurrences larger than five in the keywords analysis were “ovarian cancer”, “pancreatic cancer”, and “thyroid cancer”. After merging keywords with similar meanings, keyword co-occurrence analysis is shown in Figure 7. The big yellow node in Figure 7 is an emerging and well-developed area including deep learning, indicating that in the future, deep learning will still maintain a high research interest.

### 3.7. Co-Citation References

**Figure 8:**
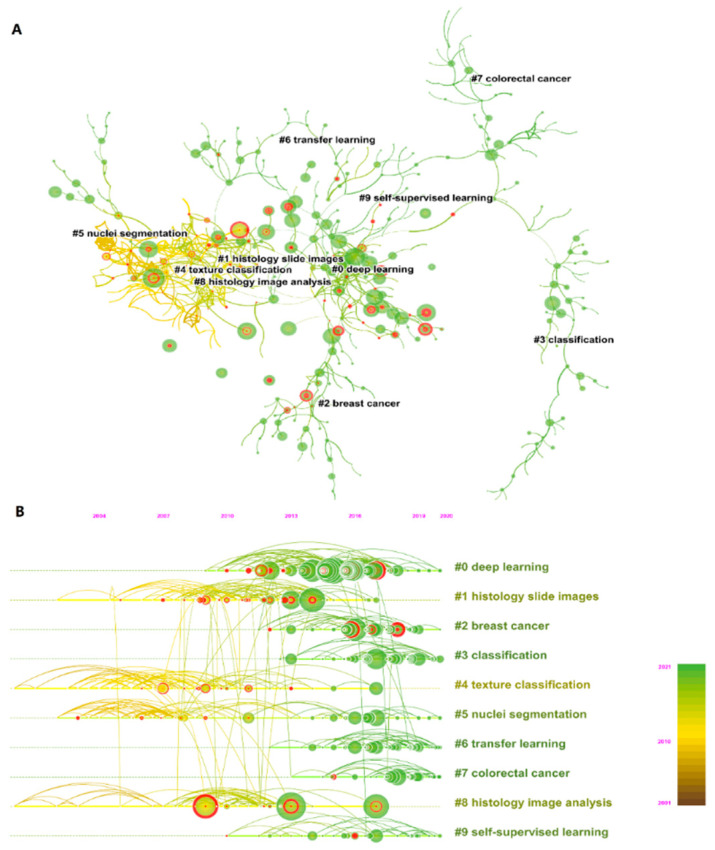
(**A**) The co-citation map of reference. (**B**) Timeline visualization of co-citation map. The label selection method was the log-likelihood ratio. Lines indicate reference relations, and color of lines and circles from yellow to green represent the years from 2001 to 2021. Red circles indicate the burst citation, which means that the number of citations to the publication increased rapidly, lasting for multiple years or a single year. The vertical direction from top to bottom represents the cluster from large to small, the largest cluster is shown on the top with label “#0”. The horizontal direction represents the timeline from 2001 to 2021. A node indicates a publication. The larger node size, the more times co-cited.

We summarize the top 10 most cited references in Table 1. Ronneberger et al. have the highest total citation frequency with 358 citations, and their proposed U-Net is widely used in medical image segmentation [44]. Modularity Q and the weighted mean Silhouette S of the co-citation network (Figure 8A) were 0.9149 and 0.9556, respectively, showing a good clustering effect and clear topics of the co-citation network. We selected the topics of the top 10 clusters and plotted a timeline map [45,46] (Figure 8B) to find out the development trend in the past years. Deep learning is the largest cluster in the period from 2010 to 2020. Clusters 2 and 7 show that recent studies cited on breast cancer and colorectal cancer have increased sharply. Clusters 3, 4, 5, 6, and 9 (i.e., classification, texture classification, nuclei segmentation, transfer learning, and self-supervised learning, respectively) have been the research hotspots of AI in HI analysis in recent years. Texture classification (cluster 4) and nuclei segmentation (cluster 5) were studied earlier, generally based on traditional machine learning or feature engineering [47,48,49,50]. Transfer learning and self-supervised learning are emerging methods from 2010 to 2021 [51,52,53,54]. The detail about these methods is in the Discussion section.

## 4. Discussion

In this study, we conducted a bibliometric analysis of 2844 documents to understand the research status, research hotspots, and research development trends in the application of AI in HI analysis. In the past 20 years, the number of publications has increased year after year, especially sharply in the past five years. Breast cancer, prostate cancer, colorectal cancer, and lung cancer are the four most studied cancer types. The USA is the most influential and productive country with far more citations than any other country, and it has collaborated extensively with multiple countries. American researchers and institutions have carried out artificial intelligence pathology research for the longest and have published many high-quality articles. Meanwhile, countries and institutions should strengthen cooperation with each other to promote advanced research and interdisciplinary frontier research. 

With comprehensive keyword co-occurrence analysis and reference co-citation analysis, we summarize the challenges and main directions of AI in HI analysis. On the one hand, classification based on deep learning and nuclei segmentation based on deep learning may be the two main research directions at present. Usually, deep learning methods require large datasets with annotation to reduce overfitting and improve generalization capability. On the other hand, articles related to transfer learning and self-supervised learning were published and updated rapidly, indicating these two directions could be potential hotspots in the future.

Classification based on deep learning. Classification is aimed at diagnosing cancer and identifying cancerous tissue. AI methods will develop the formation of an objective and accurate cancer identification process in cancer diagnosis. In general, the input image size is much smaller than the histopathological image in most mainstream deep network architecture for classification tasks. A scanned histopathological image usually contains billions of pixels, needing image preprocessing into patches with appropriate sizes as the input for the deep network. A scanned histopathological image is divided into non-overlapping patches of the same size, and in this process, may generate thousands of image patches. Datasets will be manually annotated by pathologists at the patch level. The deep learning model will be trained on the above dataset to learn to extract the relevant features, guided by annotations. The more annotated the data, the higher classification performance of the deep learning model. For high-precision recognition models, deep learning methods are comparable to professional pathologists [45,61], but it is still difficult for most pathological models to form an effective comparison with pathologists. The biggest challenge of current classification problems is that training accurate classification models requires large datasets with physician annotations, which are often difficult to obtain. To deal with this problem, a commonly used practice at this stage is to expand the data by augmentation methods (e.g., color changes, random cropping, etc.). Collecting large datasets with annotations [62,63] and using pre-training methods such as transfer learning [64,65,66] and self-supervised learning [67,68] are effective methods to improve classification accuracy. In addition, the emergence of automated annotation procedures [69,70,71] provides an alternative to manual annotations by pathologists to reduce the requirements of professionals for data annotation.

Nuclei segmentation. Nuclear segmentation analyzes the features of cancer tissue and evaluates grade and prognosis. Nuclear features are significant in histopathology, as pathologists may diagnose the grading of patients or assess prognosis based on cell morphology and structure. For example, mitosis is closely related to breast cancer grading [72]. Nuclear density, size, and other indicators have a significant relationship with cancer prognosis [73]. The segmentation of nuclei from tissue images to extract nuclei features provides a premise for future analysis [74,75]. In a nuclei segmentation dataset, some representative regions of interest (ROIs) are selected from HI images by the pathologist, annotating the cell/nuclei contours and cell/nuclei types using annotation software. Typically, a single ROI contains hundreds of cells and several cell types, which takes great effort to annotate accurately segmentation ground truth. In nuclei segmentation methods, each pixel of the input image will be predicted as the probability of belonging to the cell/nuclei as output in the deep learning model. Pixel probabilities larger than a certain probability threshold are considered to belong to cell/nuclei region. One of the challenges in nuclei segmentation is the overlapping of nuclei and the gathered nuclei. Nuclei annotation is at the cell instance level, which is more difficult to annotate. Moreover, there are few publicly available datasets, leading to the segmentation method being difficult to expand between different datasets [76]. 

Transfer learning. Currently, there are several large datasets with complete annotation in some downstream tasks such as image classification [61], segmentation [77], and object detection [78,79]. In HI, datasets are usually annotated for a specific downstream task in a specific cancer or tissue [80,81,82], hindering study on other cancer types which other researchers are interested in. Transfer learning is a method to leverage knowledge from a source domain to improve the learning performance in a target domain [83]. Transfer learning can increase the performance and robustness of relatively small data in downstream analysis when it is difficult to obtain large datasets with annotation. In HI analysis, transfer knowledge gained from other large datasets to target the cancer domain can improve the performance of downstream tasks [54,84,85,86]. The difficulty of histopathological transfer learning is that most studies transfer the ImageNet dataset [61] to the medical data domain [87]. The ImageNet dataset contains natural objects, while the HI dataset contains repeated organizational structures and texture features. There are significant differences in the distribution of features and resolution between the two data formats. Based on the currently available large datasets of histopathology, suitable transfer learning methods could improve the performance of downstream tasks [87,88,89].

Self-supervised learning. Self-supervised learning is a method that obtains a representation of a dataset without annotation data, unsupervised to find common features among images from unlabeled datasets [90]. Self-supervised learning builds pretext tasks to learn a good representation of the inherent features in the training process, and fine-tunes downstream tasks using labeled data [91]. The pretext tasks of HI are generally constructed through contextual, multi-resolution, and semantic features [51,92], and the downstream tasks are analyzed by pre-training and fine-tuning. Effective features of HI are extracted using appropriate pretext tasks using prior information possessed in HI [93]. Self-supervised learning excavates image-learning semantic information from the image itself, which is the development direction of AI in medicine and of HI analysis in the future [94]. Learning how to design an appropriate pretext task for HI and obtaining general features are the key points in the application of self-supervised learning in HI.

### Limitations

This study represents the first bibliometric analysis of the application of AI on HI that includes comprehensive cancer types. This study also has limitations. At present, only the literature in the WOSCC database has been analyzed; documents in other databases may have been missed. To prevent the influence of automatic keyword selection by WOSCC, only the keywords provided by the authors were used in the keyword analysis, which may lead to the omission of some keywords. Recently published publications may be biased due to low citation rates.

In future studies, researchers can study bibliometric analysis in depth for the specific cancer type that is most studied, according to Table S5. They should also include more databases to obtain more comprehensive publications and should manually select all the keywords to obtain a more accurate keyword analysis.

## 5. Conclusions

In summary, AI has become an important research method for HI analysis. Breast cancer, prostate cancer, colorectal cancer, and lung cancer are the most studied cancer types. Classification and nucleus segmentation based on deep learning may continue to be research hotspots for a long time. Transfer learning and self-supervised learning are likely the development trends of future research. In general, this study provides some insights into the structure and trends in the field of AI HI analysis.

## Figures and Tables

**Table 1:** Top 10 most co-cited publications of AI in HI.

Rank	Title	Author	Count	Year	Reference
1	U-Net: Convolutional Networks for Biomedical Image Segmentation	Ronneberger, Olaf	191	2015	[44]
2	Diagnostic Assessment of Deep Learning Algorithms for Detection of Lymph Node Metastases in Women With Breast Cancer	Bejnordi, Babak Ehteshami	164	2017	[55]
3	Deep Residual Learning for Image Recognition	He, KM	132	2016	[56]
4	Very Deep Convolutional Networks for Large-Scale Image Recognition.	Simonyan, Karen	129	2014	[57]
5	Classification and mutation prediction from non-small cell lung cancer histopathology images using deep learning	Coudray, Nicolas	119	2018	[7]
6	Deep learning	LeCun, Yann	118	2015	[58]
7	Deep learning for digital pathology image analysis: A comprehensive tutorial with selected use cases	Janowczyk, Andrew	116	2016	[59]
8	Mitosis Detection in Breast Cancer Histology Images with Deep Neural Networks	Cireşan, DC	115	2013	[60]
9	ImageNet classification with deep convolutional neural networks	Krizhevsky, Alex	112	2012	[61]
10	Dermatologist-level classification of skin cancer with deep neural networks	Esteva, Andre	110	2017	[37]

## Data Availability

Not applicable.

## Supplementary Materials

The following supporting information can be downloaded at: https://www.mdpi.com/article/10.3390/ijerph191811597/s1. Table S1: Top 10 most productive countries of AI in HI from 2001 to 2021; Table S2: Top 10 most productive institutions of AI in HI from 2001 to 2021; Table S3: Top 10 most productive authors of AI in HI from 2001 to 2021; Table S4: Top 10 journals publishing research on AI in HI from 2001 to 2021; Table S5: Top 10 most frequently used keywords for methods and cancer types in publications of AI in HI.
