# Supplementary material for: Global Research Trends of Artificial Intelligence on Histopathological Images: A 20-Year Bibliometric Analysis
Source: Int J Environ Res Public Health. 2022 Sep 15;19(18):11597. doi: 10.3390/ijerph191811597 (PMC9517580; doi:10.3390/ijerph191811597)
Supplement: Supplementary file 1 [file ijerph-19-11597-s001.zip › ijerph-1874582-supplementary.pdf]

Table S1. Top 10 most productive countries of AI in HI from 2001 to 2021.

Rank	Country/Region	Documents	Citations	TLS[1]
1	USA	937	23010	731
2	China	550	6631	316
3	India	263	2318	135
4	England	229	5034	360
5	Germany	219	4592	311
6	Canada	166	2352	157
7	Netherlands	118	7548	201
8	Turkey	99	1154	52
9	Italy	98	1413	157
10	France	98	3301	151

[1]Total link strength (TLS) indicates the strength between countries. The higher the value, the higher the total cooperation intensity.

Table S2. Top 10 most productive institutions of AI in HI from 2001 to 2021.

Rank	Institutions	Country/Region	Documents	Citations	TLS
1	Emory University	USA	55	1319	103
2	The University of Warwick	UK	54	1538	74
3	Case Western Reserve University	USA	53	2872	93
4	Radboud University Nijmegen	Netherlands	49	6316	67
5	University of Toronto	Toronto	46	776	52
6	Harvard Medical School	USA	46	721	91
7	Stanford University	USA	43	5423	40
8	The State University of New Jersey	USA	39	1393	22
9	Beihang University	China	36	806	47
10	The Ohio State University	USA	35	553	44

Table S3. Top 10 most productive authors of AI in HI from 2001 to 2021.

Rank	Author	Country/Region	Documents	Citations
1	Madabhushi, Anant	USA	72	3400
2	Rajpoot, Nasir	England	45	1285
3	Van Der Laak, JAWM	Netherlands	31	5963
4	Bilgin, Gokhan	Turkey	29	116
5	Tomaszewski, John	USA	24	1077
6	Litjens, Geert	Netherlands	23	5592
7	Yang, Lin	USA	21	637
8	Saltz, Joel	USA	20	631
9	Sarder, Pinaki	USA	18	192
10	Janowczyk, Andrew	Switzerland	17	323

Table S4. Top 10 journals publishing research on AI in HI from 2001 to 2021.

Rank	Journals	Documents	Citations	2020 IF[1]
1	Scientific Reports	84	1375	4.38
2	Medical Image Analysis	59	5926	8.545
3	IEEE Transactions on Medical Imaging	48	2832	10.048
4	IEEE Access	46	451	3.367
5	Cancers	44	325	6.639
6	Computers in Biology and Medicine	41	420	4.589
7	IEEE Journal of Biomedical and Health Informatics	37	172	5.772
8	Frontiers in Oncology	37	459	6.244
9	Computerized Medical Imaging and Graphics	26	487	4.79
10	PLOS One	26	555	3.24

[1]IF is impact factor in 2020 from Web of Science.

Table S5. Top 10 most frequently used keywords for methods and cancer types in publications of AI in HI.

Rank	Methods	Occurrences	Cancer types	Occurrences
1	Deep learning	657	Breast cancer	298
2	Convloutional nerual network	445	Prostate cancer	90
3	Machine learning	298	Colon cancer	55
4	Image segmentation	258	Lung cancer	45
5	Classification	240	Cervical cancer	26
6	Transfer learning	108	Gastric cancer	21
7	Feature extraction	100	Oral cancer	14
8	Support vector machine	76	Bladder cancer	9
9	Nuclei segmentation	63	Head and neck cancer	9
10	Nerual network	41	skin cancer	9
